# The Gut Microbiota in Liver Transplantation Recipients During the Perioperative Period

**DOI:** 10.3389/fphys.2022.854017

**Published:** 2022-04-01

**Authors:** Zhiyong Lai, Zongkun Chen, Anhong Zhang, Zhiqiang Niu, Meng Cheng, Chenda Huo, Jun Xu

**Affiliations:** ^1^ Department of Hepatobiliary and Pancreatic Surgery and Liver Transplant Center, the First Hospital of Shanxi Medical University, Taiyuan, China; ^2^ Shanxi Medical University, Taiyuan, China

**Keywords:** chronic liver disease, liver transplantation, gut, microbial composition, alpha diversities, beta diversities

## Abstract

**Background:** Chronic liver disease is a global problem, and an increasing number of patients receive a liver transplant yearly. The characteristics of intestinal microbial communities may be affected by changes in the pathophysiology of patients during the perioperative.

**Methods:** We studied gut fecal microbial community signatures in 37 Chinese adults using 16S rRNA sequencing targeting V3-V4 hypervariable regions, with a total of 69 fecal samples. We analyzed the Alpha and Beta diversities of various groups. Then we compared the abundance of bacteria in groups at the phylum, family, and genus levels.

**Results:** The healthy gut microbiota predominantly consisted of the phyla Firmicutes and Bacteroidestes, followed by Proteobacteria and Actinobacteria. Compared with healthy people, due to the dominant bacteria in patients with chronic liver disease losing their advantages in the gut, the antagonistic effect on the inferior bacteria was reduced. The inferior bacteria multiplied in large numbers during this process. Some of these significant changes were observed in bacterial species belonging to *Enterococcus*, *Klebsiella*, and *Enterobacter*, which increased in patients’ intestines. There were low abundances of signature genes such as *Bacteroides*, *Prevotella*, and *Ruminococcus*. *Blautia* and *Bifidobacterium* (considered probiotics) almost disappeared after liver transplantation.

**Conclusion:** There is an altered microbial composition in liver transplantation patients and a distinct signature of microbiota associated with the perioperative period.

## Introduction

Around 2 million deaths annually are attributable to liver disease worldwide: 1 million dues to cirrhosis and 1 million due to viral hepatitis and hepatocellular carcinoma (GBD 2017 Cirrhosis Collaborators, 2020). Cirrhosis is the 11th most common cause of death and the third leading cause of death among people aged 45–64 years. With liver cancer, cirrhosis accounts for 3.5% of global deaths (Asrani et al., 2019). Liver disease affects 1.3 billion people worldwide. Chronic liver disease and cirrhosis cause 44,000 deaths in the United States and 2 million deaths worldwide every year (Mokdad et al., 2014; Tapper et al., 2018). Asia is one of the regions with the highest prevalence of liver diseases. In China alone, 300 million people are affected, making the country a global leader in the prevalence of liver diseases. The incidence of cirrhosis is the leading cause of associated mortality and morbidity. The annual mortality rate is more than 1 million, which has increased in some countries (Rowe, 2017; Wang et al., 2014).

Cirrhosis is the terminal phase of liver disease. In the absence of liver transplantation, patients face dire outcomes. Innovations in surgical equipment and the development of new immunosuppressants have increased the success rate of liver transplantation and prolonged postoperative survival. Nevertheless, in all patients with liver disease, the proportion of malnutrition is as high as 25%–56% and 65%–90% in patients with advanced liver cirrhosis (Yao et al., 2018). There are many factors leading to malnutrition, including nausea, anorexia, alterations in taste receptors, loss of appetite, reduced oral intake of energy and protein, increased basal metabolic rate, unnecessary fasting, and restricted diet.

Under the influence of many factors, the intestinal microbiota of patients with liver disease is significantly different from that of ordinary people. The most dominant bacterial phyla in the human gut are Firmicutes, Bacteroidetes, Actinobacteria, and Proteobacteria, and the most recorded bacterial genera are *Bacteroides*, *Clostridium*, *Peptococcus*, *Bifidobacterium*, *Eubacterium*, *Ruminococcus*, *Faecalibacterium*, and *Peptostreptococcus* (Shapira, 2016). It is well known that human intestinal microbiota is a substantial bacterial library, and there are trillions of microorganisms in 1 G of feces (Turnbaugh et al., 2009). Throughout evolutionary history, humans have developed a symbiotic relationship with bacteria, which protect the gut by providing the host with essential vitamins and nutrients (Schnabl et al., 2014). The gut microbiota performs several essential functions, including protection from pathogens by colonizing mucosal surfaces and creation of various antimicrobial substances, enhancing the immune system, playing a vital role in digestion and metabolism, controlling epithelial cell proliferation and differentiation, modifying insulin resistance, and affecting its secretion, influencing brain-gut communication, and thus affecting the mental and neurological functions of the host. In brief, the gut microbiota plays a significant role in maintaining normal gut physiology and health (Zheng et al., 2019; Kelly et al., 2015; Mills et al., 2019; Rothschild et al., 2018; Wiley et al., 2017).

The balance between microorganisms parasitic in the human intestinal tract may be destroyed, resulting in adrenoleukodystrophy, inflammatory bowel disease, infections, autism, Parkinson’s disease, and cancer. However, 20%–60% of bacteria in the human body cannot be cultured with current methods (Kwong et al., 2021). Sequence analysis of 16S rRNA identified several hundred bacterial species in the intestinal ecosystem, most of which cannot be cultured.

Therefore, in this study, 16S rRNA amplicon sequencing was used to analyze the diversity of intestinal microbiota in patients undergoing liver transplantation, to compare the differences of intestinal microbiota between patients undergoing liver transplantation and healthy people. Systematic bacterial profile analysis was carried out at the highest taxonomic level (L2) and the lowest possible in this research method (L6) to obtain a general picture and a detailed analysis of differences in the gut microbiota composition. We explored the impact of intestinal microbiota on patients undergoing liver transplantation and its possible mechanisms to provide a theoretical basis for fecal bacterial transplantation in the future.

## Materials and Methods

### Study Population and Sample Collection

The gut fecal microbial community signatures of 37 Chinese adults were studied for 69 fecal samples. Stool samples were collected from the First Hospital of Shanxi Medical University in 2020–2021. The entire study design and procedures involved were established following the Declaration of Helsinki. Written informed consent forms were signed before the time of sample collection. The Ethics Committee of the First Hospital of Shanxi Medical University approved the protocols. We excluded participants suffering from any symptoms of constipation, bloody stool, diarrhea, or other gastrointestinal disease and those who were administered antibiotics (oral or injectable) in the previous 3 months. In addition, all liver transplant patients in the study were used the same Immunosuppressant treatment regimen, including Tacrolimus, Sirolimus, Mycophenol ethyl ester, and Methylprednisolone, and all patients were used Cefoperazone sodium sulbactam sodium to prevent infection during the perioperative period. Furthermore, abdominal drainage fluid were cultured daily after liver transplantation, and the Antibiotic treatment strategy was adjusted according to the results of bacterial culture of drainage fluid. All stool samples were collected within 4 h. Stool samples were collected in sterile containers provided to the volunteers and were stored at −80°C. Sampling was performed using all standard protocols and regulations. Our analysis was conducted on a total of 69 fecal samples subdivided as follows: before liver transplantation (BLT, 16 samples); liver transplantation 1 week (LT1W, 16 samples); liver transplantation 2 weeks (LT2W, 16 samples); and control group (CG, 21 samples).

### Sampling and DNA Extraction

Upon collection, fecal samples were frozen at −80°C immediately upon collection and stored for later use. At the beginning of the experiment, 180–200 mg of each sample was weighed out and transferred to a 2-ml centrifuge tube, which was then placed on ice. According to the manufacturer’s instructions, DNA was extracted from the samples using the FastDNA^®^ Spin Kit for Soil (MP Biomedical, LLC, catalog 116560-200). We used Nanodrop to measure the extracted nucleic acid concentrations and stored samples at −80°C.

### 16S rRNA Gene Amplicon Sequencing

For sequencing, isolated fecal DNA was used as a template for amplification, and the V4 region of 16S rRNA was amplified by performing PCR assays using the universal bacterial primer set 342F (5′-CCT​ACG​GGA​GGC​AGC​AG-3′) and 806R (5′-GGACTACHVGGGTWTCTAAT-3′). The PCR reaction triplicate 50 μL mixture contained 5 μL of 10X Taq DNA polymerase PCR buffer, 1 μL of dNTP mix, 0.5 μL of Taq DNA polymerase, 2 μL of each primer (10 μM), and 10 ng/μL DNA. The reaction steps were as follows: initialized at 94°C for 5 min, 32 cycles at 94°C for 30 s, 53°C for 30 s and 72°C for 1 min and final extension at 72°C for 5 min. According to the manufacturer’s instructions, the resulting PCR products were purified *via* separation on a 2% agarose gel, followed by DNA isolation using the GeneJETTM Gel Extraction Kit (Thermo Scientific). According to the manufacturer’s instructions, the purified DNA was quantified using Ion Plus Fragment Library Kit (Life Technologies) and generated sequencing libraries. The partial 16S rRNA genes were sequenced on an Ion S5 Sequencing platform (Sun et al., 2017).

### Bioinformatics Analysis and Statistical Processing

16S rRNA sequencing data were processed using QIIME 2 software. After the original data were sequenced off the machine, FastQC software preprocessed the data, deleted incorrect sequences, and had quality control. Then, the DADA2 and Deblur method was used to perform denoising, remove low-quality sequences, short sequences, and chimera sequences in the data. This process retained 40–60% sequences (the length of 300–600 bp) and generated a feature table and representative sequences for downstream analysis. In the case of non-normal distribution, the Kruskal-Wallis analysis of variance was used to calculate variability between the four study groups. The Wilcoxon rank-sum test was used to analyze variability between the two study groups. Alpha and Beta diversities were evaluated using QIIME software, and differences were significant at *p* < 0.05.

Usearch was used to bin reads into operational taxonomic units (OTUs) with a 0.97 identity cut-off. Samples with more sequenced reads had more observed OTUs (Spearman’s rank correlation analysis) when all reads were binned into OTUs. Thus, we randomly chose reads with the same number (10,000) and then identified representative OTU reads. Finally, we mapped all randomly chosen reads against representative OTU reads to obtain the OTU composition of all samples. Diversity and richness calculation. Shannon, Simpson, and invsimpson indices were calculated using the “vegan” package in R with a normalized OTU matrix. The observed species, Chao1, and ICE indices were calculated using the “fossil” package in R with a non-normalized OTU matrix. The Hellinger distance was calculated using the “topicmodels” package in R. The JSD distance was calculated using a custom R script provided by the European Molecular Biology Laboratory enterotyping tutorial (http://enterotype.embl.de/enterotypes.html) (Sun et al., 2017).

Alpha diversity index (Shannon Diversity Index, Observed Species, Chao 1) was calculated and displayed using QIIME2 (2020.6.0) and R software (v 4.0.2). To evaluate the sequencing depth and status of sampling, the coverage of GOOD was calculated, and rarefaction curves were constructed. Based on OTU abundance and system development branch length, unweighted UniFrac distances were used in QIIME to calculate Beta diversity between samples. Principal coordinates analysis (PCoA) was performed using vegan (v 2.4–4), ggplot2 (v 3.2), and stats (v 3.6.2) packages. Alpha diversity analysis was performed using R (v 4.0.2, 2020.6), with Wilcoxon Rank-Sum test, Kruskal-Wallis Rank-Sum test, and Spearman correlation analysis. Permutation multivariate analysis of variance (PERMANOVA) and the Wilcoxon Rank-Sum test were used to test the significance of the community composition and structural differences among the groups.

### Clusters of Orthologous Genes and Pathway Profiles

COG profiles were constructed using PICRUSt (v 2.3.0-Beta) genome prediction software. First, our custom representative OTU reads were aligned against the Greengenes v.13.5 database 16S rRNA Fasta reference database, and the abundances of representative OTU reads from the same 16S rRNA reference were summed. The reference profile normalized by the 16S rRNA copy number was used to predict the COG profile, and PICRUSt2 software was used to determine the abundance of COG pathways and modules. Finally, R software calculated the top 20 taxonomic phyla and family abundances.

## Results

### Quality Control

The gut microbiota signatures of 37 Chinese adults were studied using 16S rRNA sequencing targeting V3-V4 hypervariable regions. FastQC was used to control the length and quality of sequencing data. If the sequencing data were V3 and V4 regions, the data with a length between 300 and 600 nt were retained, and if the sequencing data were V3 regions, the data with a length between 100 and 300 nt were retained. The sequences with similarities above 97% were divided into an operational taxonomic unit (OTU).

**FIGURE 1:**
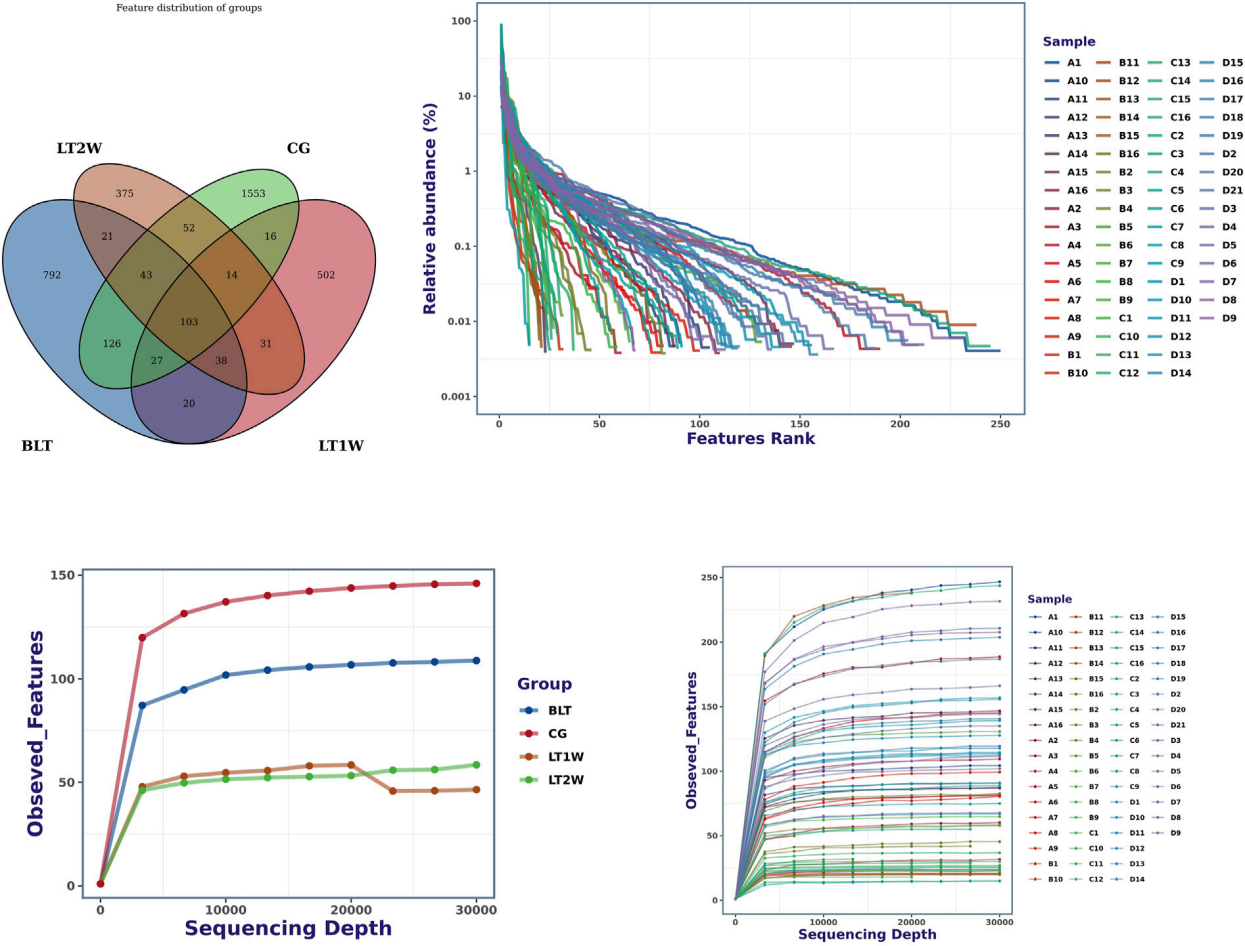
Venn diagram and rank abundance curves for all groups at the OTU level. A = BLT, B = LT1W, C = LT2W, and D = CG. Species accumulation curves were used to determine the sampling depth. The sample diversity and degree of uniformity were measured using the rank abundance curve method.

According to the results of OTUs analysis, we obtained a total of 3713 OTUs, including 1553 OTUs unique to CG group, 792 OTUs unique to BLT group, 502 OTUs unique to LT1W group and 375 OTUs unique to LT2W group (Figure 1). The top 10 OTUs unique to CG: OTU89 (d__Bacteria; p__Bacteroidota; c__Bacteroidia; o__Bacteroidales; f__*Bacteroidaceae*; g__*Bacteroides*), OTU101 (d__Bacteria; p__Firmicutes; c__Bacilli; o__Erysipelotrichales; f__Erysipelotrichaceae; g__Dubosiella; s__Uncultured_bacterium), OTU119 (d__Bacteria; Alloprevotella), OTU187 (d__Bacteria; p__Bacteroidota; c__Bacteroidia; o__Bacteroidales; f__*Prevotellaceae*; g__Prevotella), OTU213 (d__Bacteria; p__Bacteroidota; c__Bacteroidia; o__Bacteroidales; f__*Bacteroidaceae*; g__*Bacteroides*; s__*Bacteroides*_stercoris); The top 10 OTUs unique to BLT group:OTU41 (d__Bacteria; p__Bacteroidota; c__Bacteroidia; o__Bacteroidales; f__*Bacteroidaceae*; g__*Bacteroides*; s__*Bacteroides*_plebeius), OTU63 (d__Bacteria; p__Fusobacteriota; c__Fusobacteriia; o__Fusobacteriales; f__Fusobacteriaceae; g__*Fusobacterium*; s__*Fusobacterium*_mortiferum), OTU118 (d__Bacteria; p__Bacteroidota; c__Bacteroidia; o__Bacteroidales; f__*Prevotellaceae*; g__Prevotella), OTU147 (d__Bacteria; p__Firmicutes; c__Clostridia; o__Oscillospirales; f__Ruminococcaceae; g__Faecalibacterium), OTU162 (d__Bacteria; p__Verrucomicrobiota; c__Verrucomicrobiae; o__Verrucomicrobiales; f__Akkermap__Firmicutes; c__Clostridia; o__Oscillospirales; f__Ruminococcaceae; g__CAG-352; s__uncultured_bacterium), OTU120 (d__Bacteria; p__Bacteroidota; c__Bacteroidia; o__Bacteroidales; f__*Bacteroidaceae*; g__*Bacteroides*; s__*Bacteroides*_coprophilus), OTU146 (d__Bacteria; p__Firmicutes; c__Negativicutes; o__Veillonellales-Selenomonadales; f__Selenomonadaceae; g__Megamonas), OTU151 (d__Bacteria; p__Bacteroidota; c__Bacteroidia; o__Bacteroidales; f__*Bacteroidaceae*; g__*Bacteroides*), OTU158 (d__Bacteria; p__Proteobacteria; c__Gammaproteobacteria; o__Enterobacterales; f__Enterobacteriaceae; g__Escherichia-Shigella), OTU173 (d__Bacteria; p__Bacteroidota; c__Bacteroidia; o__Bacteroidales; f__*Prevotellaceae*; g__ nsiaceae; g__Akkermansia), OTU167 (d__Bacteria; p__Firmicutes; c__Clostridia; o__Oscillospirales; f__Ruminococcaceae; g__Faecalibacterium), OTU169 (d__Bacteria; p__Bacteroidota; c__Bacteroidia; o__Bacteroidales; f__*Bacteroidaceae*; g__*Bacteroides*; s__*Bacteroides*_stercoris), OTU183 (d__Bacteria; p__Bacteroidota; c__Bacteroidia; o__Bacteroidales; f__*Prevotellaceae*; g__Prevotella), OTU199 (d__Bacteria; p__Bacteroidota; c__Bacteroidia; o__Bacteroidales; f__*Prevotellaceae*; g__Prevotella), OTU218 (d__Bacteria; p__Firmicutes; c__Clostridia; o__Oscillospirales; f__Ruminococcaceae; g__Faecalibacterium); The top 10 OTUs unique to LT1W group: OTU73 (d__Bacteria; p__Proteobacteria; c__Gammaproteobacteria; o__Enterobacterales; f__Enterobacteriaceae; g__Escherichia-Shigella), OTU108 (d__Bacteria; p__Firmicutes; c__Bacilli; o__Lactobacillales; f__*Enterococcaceae*; g__*Enterococcus*), OTU154 (d__Bacteria; p__Proteobacteria; c__Gammaproteobacteria; o__Pseudomonadales; f__Moraxellaceae; g__*Acinetobacter*; s__*Acinetobacter*_baumannii), OTU170 (d__Bacteria; p__Firmicutes; c__Bacilli; o__Lactobacillales; f__*Lactobacillaceae*; g__*Lactobacillus*), OTU174 (d__Bacteria; p__Actinobacteriota; c__Actinobacteria; o__Bifidobacteriales; f__*Bifidobacteriaceae*; g__Bifidobacterium; s__Bifidobacterium_breve), OTU200 (d__Bacteria; p__Firmicutes; c__Negativicutes; o__Veillonellales-Selenomonadales; f__Veillonellaceae; g__Veillonella), OTU245 (d__Bacteria; p__Actinobacteriota; c__Coriobacteriia; o__Coriobacteriales; f__Atopobiaceae; g__Olsenella), OTU265 (d__Bacteria; p__Actinobacteriota; c__Actinobacteria; o__Bifidobacteriales; f__*Bifidobacteriaceae*; g__Bifidobacterium; s__Bifidobacterium_breve), OTU270 (d__Bacteria; p__Firmicutes; c__Negativicutes; o__Veillonellales-Selenomonadales; f__Veillonellaceae; g__Veillonella), OTU280 (d__Bacteria; p__Firmicutes; c__Clostridia; o__Peptostreptococcales-Tissierellales; f__Peptostreptococcales-Tissierellales; g__Parvimonas); The top 10 OTUs unique to LT2W group: OTU29 (d__Bacteria; p__Proteobacteria; c__Gammaproteobacteria; o__Enterobacterales; f__Enterobacteriaceae), OTU62 (d__Bacteria; p__Proteobacteria; c__Gammaproteobacteria; o__Enterobacterales; f__Yersiniaceae; g__*Serratia*),OTU74 (d__Bacteria; p__Proteobacteria; c__Gammaproteobacteria; o__Enterobacterales; f__Yersiniaceae; g__*Serratia*), OTU79 (d__Bacteria; p__Proteobacteria; c__Gammaproteobacteria; o__Enterobacterales; f__Yersiniaceae; g__*Serratia*), OTU178 (d__Bacteria; p__Proteobacteria; c__Gammaproteobacteria; o__Enterobacterales; f__Enterobacteriaceae), OTU196 (d__Bacteria; p__Proteobacteria; c__Gammaproteobacteria; o__Enterobacterales; f__Enterobacteriaceae; g__*Klebsiella*), OTU197 (d__Bacteria; p__Proteobacteria; c__Gammaproteobacteria; o__Enterobacterales; f__Enterobacteriaceae; g__*Enterobacter*), OTU228 (d__Bacteria; p__Proteobacteria; c__Gammaproteobacteria; o__Enterobacterales; f__Enterobacteriaceae), OTU231 (d__Bacteria; p__Proteobacteria; c__Gammaproteobacteria; o__Enterobacterales; f__Enterobacteriaceae; g__*Enterobacter*), OTU250 (d__Bacteria; p__Proteobacteria; c__Gammaproteobacteria; o__Enterobacterales; f__Enterobacteriaceae).

We counted the number of sequences contained in all OTUs in each sample, sorted OTUs from high to low according to abundance, and generated rank abundance curves (Figure 1). In terms of horizontal axis distribution, CG was relatively wider than the other three groups, suggesting that the species distribution of CG is more abundant; From the vertical axis distribution, the curves distribution of CG were gentle downward compared with the other three groups, suggesting that the uniformity of species composition in the healthy control group is relatively high. LT1W and LT2W groups were considerably narrow on the horizontal axis, suggesting that the species abundance of the two groups is low, and the vertical axis was steep, suggesting that the species distribution uniformity is poor.

### Diversity Analysis

#### Alpha Diversity Analysis

**FIGURE 2:**
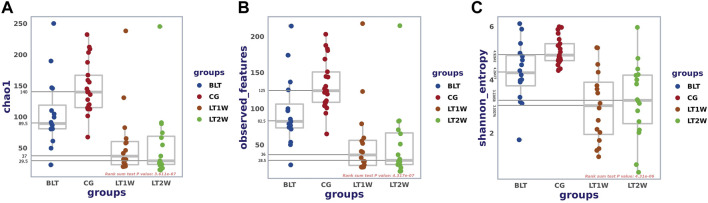
Alpha diversity between liver transplantation patients. Comparison of Boxplots depicting chao1 **(A)**, observed-features **(B)**, and Shannon index **(C)**. Diversity among CG (*n* = 21), BLT (*n* = 16), LT1W (*n* = 16), and LT2W (*n* = 16) groups.

Alpha diversity was quantified using the Chao1 index, observed features, and Shannon diversity indexes, which relate OTU richness and evenness and the total number of observed species. Evaluation of taxonomic pattern of gut microbiota in liver transplantation patients showed that the alpha-diversity calculated using chao1 index was higher in the control group, reflecting a reduction of gut microbiota diversity following liver transplantation (Figure 2). The Chao1 of CG and BLT groups were significantly higher than those of other groups, while the Chao1 of LT1W and LT2W groups was relatively low (Table 1). The species richness of CG was significantly higher than the BLT group (*p* < 0.05). The species richness in the LT1W and LT2W groups was significantly lower than that of the other two groups (*p* < 0.05). Interestingly, there was no significant difference between LT1W and LT2W groups (*p* > 0.05). As indicated in the Shannon index, there were significant differences in microbial community abundances between transplant patients and healthy controls (Table 2). As we expected, observed features showed similar results (Table 3). An evident difference in the gut microbiota was observed at all taxonomic levels between these four groups. These findings suggest that a decrease in taxonomic diversity characterizes gut microbiota in liver transplant patients. The difference between the BLT and CG groups may be related to liver disease. Chronic liver disease severely affects appetite and digestive function, and it is challenging to avoid severe ascites, the end stage of liver disease, which further affects intestinal peristalsis and causes bacterial translocation. The intestinal diversity of postoperative patients has been substantially destroyed, which may be related to antibiotics and immunosuppressants. About a week after surgery, the patients began eating by mouth, and the dosage of antibiotics began to decrease. It can be seen from the figure that the microbial richness of the LT2W group began to rise compared with the LT1W group. We believe that this is the signal that intestinal function and microecology begin to recover.

**TABLE 1:** Chao 1 index analysed with Kruskal-Wallis rank sum.

Group 1	Group 2	H	*p*-value	q-value
BLT (n = 16)	CG (n = 21)	8.130687	0.004352	0.005223
BLT (n = 16)	LT1W (n = 16)	9.558144	0.001991	0.002986
BLT (n = 16)	LT2W (n = 16)	9.559897	0.001989	0.002986
CG (n = 21)	LT1W (n = 16)	17.64841	2.66E-05	7.97E-05
CG (n = 21)	LT2W (n = 16)	19.49565	1.01E-05	6.05E-05
LT1W (n = 16)	LT2W (n = 16)	0.128337	0.720162	0.720162

**TABLE 2:** Shannon entropy analysed with Kruskal-Wallis rank sum.

Group 1	Group 2	H	*p*-value	q-value
BLT (n = 16)	CG (n = 21)	6.015038	0.014184	0.021277
BLT (n = 16)	LT1W (n = 16)	6.1875	0.012866	0.021277
BLT (n = 16)	LT2W (n = 16)	4.454545	0.034808	0.04177
CG (n = 21)	LT1W (n = 16)	17.8985	2.33E-05	6.99E-05
CG (n = 21)	LT2W (n = 16)	17.8985	2.33E-05	6.99E-05
LT1W (n = 16)	LT2W (n = 16)	0.171875	0.678451	0.678451

**TABLE 3:** Observed features analysed with Kruskal-Wallis rank sum.

Group 1	Group 2	H	*p*-value	q-value
BLT (n = 16)	CG (n = 21)	8.483148	0.003585	0.004301
BLT (n = 16)	LT1W (n = 16)	9.102588	0.002552	0.003829
BLT (n = 16)	LT2W (n = 16)	9.329862	0.002254	0.003829
CG (n = 21)	LT1W (n = 16)	17.39583	3.03E-05	9.10E-05
CG (n = 21)	LT2W (n = 16)	19.3605	1.08E-05	6.49E-05
LT1W (n = 16)	LT2W (n = 16)	0.102816	0.748476	0.748476

### Beta Diversity Analysis

**FIGURE 3:**
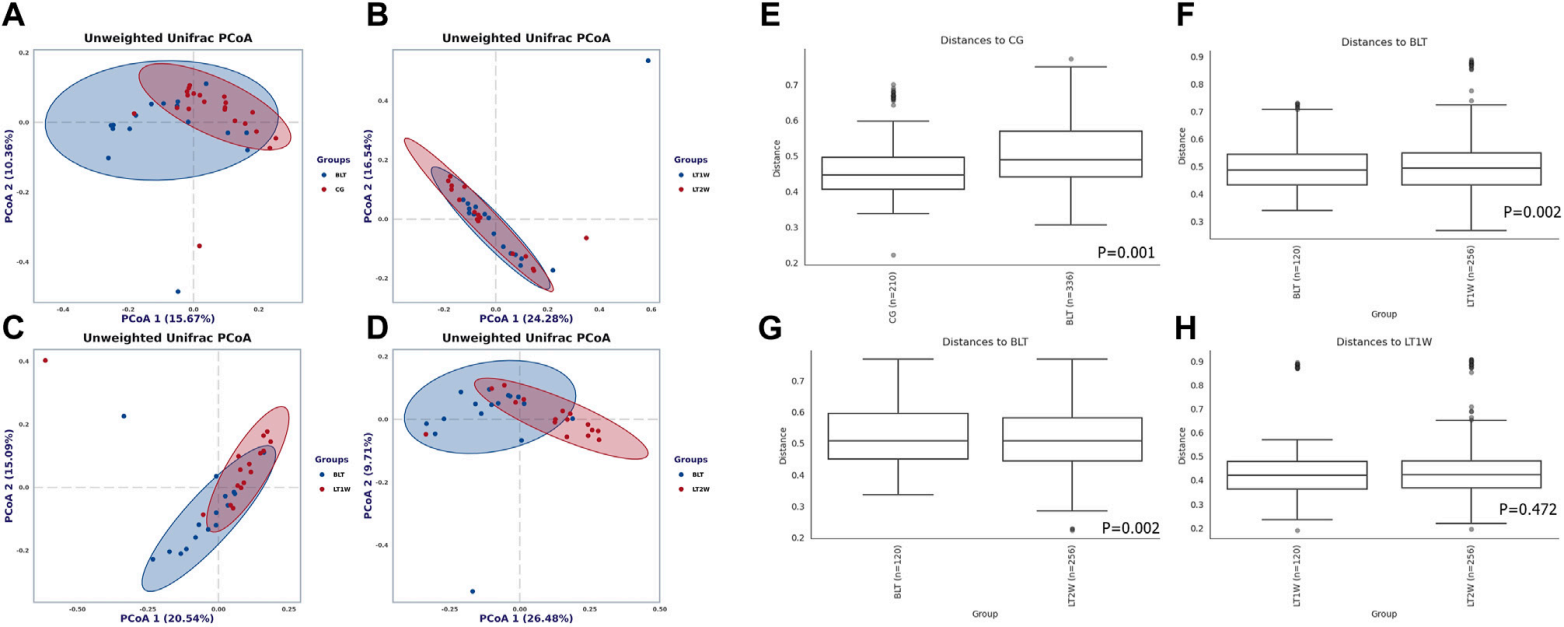
Beta diversity of gut microbial communities in liver transplantation patients and healthy participants. Principal coordinates analysis plot based on unweighted UniFrac distance. Each dot represents one sample from each group. The unweighted UniFrac patterns indicate that the liver transplantation and healthy individuals cluster separately, representing 15.67% and 10.36% **(A)**, 24.38% and 16.54% **(B)**, 20.54% and 15.09% **(C)**, and 26.48% and 9.71% **(D)** of the total variance on the *x*-axis and *y*-axis, respectively. The distance between dots on the plot indicates the degree of similarity of taxonomic composition of the samples. Charts **(E–H)** show the distance between groups and within groups, *p* < 0.05 indicating that the beta diversity between groups was significant.

Beta diversity (the degree of pair-wise similarity in the species composition among populations) was assessed using PCoA on the unweighted UniFrac metric. The calculation was based on unweighted UniFrac distance matrices constructed to demonstrate the overall dissimilarity of bacterial communities in the four groups of individuals from the Chinese population (Figure 3). According to PCoA, CG and BLT groups are highly coincident, the BLT group only partially overlaps with LT1W and LT2W groups, and the LT1W and LT2W groups almost wholly coincide. PERMANOVA demonstrates that CG and BLT groups show significant differences in Beta diversity, and the BLT group has significant in Beta diversity with the LT1W and LT2W group; by contrast, the difference of Beta diversity between LT1W and LT2W groups is not substantial (Figure 3).

### Species Composition

#### Phylum Level (L2)

**FIGURE 4:**
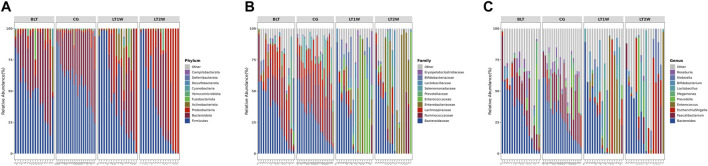
The relative abundance of gut bacteria in liver transplantation patients and healthy participants. **(A)** Relative percentage of most abundant phyla in each sample between liver transplantation patients (*n* = 16) and healthy individuals (*n* = 21). **(B)** Relative abundance of bacteria at family level in CG, BLT, LT1W, and LT2W groups. **(C)** Relative abundance of bacteria at the genus level in all groups.

At the phylum level, the Firmicutes and Bacteroidetes prevailed in all groups (Figure 4A), with relative abundances of 57.502% and 37.379% (CG), 48.834% and 40.617% (BLT group), 48.018%, and 25.594% (LT1W group), 37.168% and 26.072% (LT2W group) (Table 4). Moreover, the relative abundances of Chloroflexi, Nitrospirota, and Crenarchaeota were lower or even completely absent after liver transplantation, decreasing significantly 2 weeks after surgery. After liver transplantation, the relative abundance of Proteobacteria was significantly higher (*p* < 0.05). Microbiota of healthy volunteers was characterized by higher levels of Deferribacterota and Parabasalia, while gut microbiota from the BLT, LT1W, and LT2W groups contained none. The Actinobacteriota began to appear in the BLT group, and then its proportion increased dramatically in the LT1W group. Interestingly, Actinobacteriota almost disappeared in the LT2W group.

**TABLE 4:** The significantly different relative abundance of gut microbiota at phylum, family, and genus level. (a) The relative abundance of gut bacteria in all groups using Kruskal-Wallis rank-sum tests. (b c, d) The relative abundance of gut bacteria in double groups using Wilcoxon’s rank sum tests (b: BLT vs CG, c: BLT vs LT1W, d: BLT vs LT2W).

	CG (%)	BLT (%)	LT1W (%)	LT2W (%)	*p* Value
**Phylum (L2)**					
p__Firmicutes	57.502	48.834	48.018	37.168	*p* < 0.05ac *p* > 0.05bd
p__Bacteroidota	37.379	40.617	25.594	26.072	*p* < 0.05c *p* > 0.05abd
p__Proteobacteria	3.244	6.081	15.833	35.095	*p* < 0.05ad *p* > 0.05bc
**Family (L5)**					
f__*Lachnospiraceae*	20.249	18.010	2.679	3.667	*p* < 0.05acd *p* > 0.05d
f__Monoglobaceae	0.328	0.253	0.000	0.006	*p* < 0.05abcd
f__Burkholderiaceae	0.000	0.000	0.092	0.058	*p* < 0.05acd Na b
f__Oscillospiraceae	1.007	0.921	0.058	0.260	*p* < 0.05acd *p* > 0.05b
f__*Enterococcaceae*	0.010	0.161	17.716	7.085	*p* < 0.05ac *p* > 0.05bd
f__Butyricicoccaceae	0.262	0.125	0.000	0.043	*p* < 0.05acd *p* > 0.05b
f__Clostridiaceae	0.109	1.078	0.431	1.741	*p* < 0.05abcd
f__uncultured	0.046	0.000	0.026	0.006	*p* > 0.05ac Na bd
**Genus (L6)**					
g__Roseburia	4.055	3.516	0.024	0.256	*p* < 0.05acd *p* > 0.05b
g__Erysipelotrichaceae_UCG.003	0.344	0.811	0.000	0.027	*p* < 0.05acd *p* > 0.05b
g__Blautia	2.342	2.817	0.433	0.323	*p* < 0.05acd *p* > 0.05b
g__Lachnospira	1.091	0.639	0.003	0.033	*p* < 0.05acd *p* > 0.05b
g__Monoglobus	0.328	0.253	0.000	0.006	*p* < 0.05acd *p* > 0.05b
g__*Enterococcus*	0.010	0.161	17.716	7.050	*p* < 0.05ac *p* > 0.05bd
g__Dorea	0.313	0.262	0.000	0.041	*p* < 0.05acd *p* > 0.05b
g__[Eubacterium]_hallii_group	0.656	0.236	0.000	0.044	*p* < 0.05abcd
g__Intestinibacter	0.049	0.110	0.000	0.017	*p* < 0.05acd *p* > 0.05b
g__Romboutsia	0.344	0.240	0.000	0.002	*p* < 0.05acd *p* > 0.05b
g__Lautropia	0.000	0.000	0.029	0.058	*p* < 0.05acd NAb
g__[Eubacterium]_eligens_group	0.767	3.062	0.004	0.031	*p* < 0.05acd *p* > 0.05b
g__*Bacteroides*	27.476	34.593	22.576	18.264	*p* < 0.05acd *p* > 0.05b
g__Faecalibacterium	21.454	16.635	2.891	8.984	*p* < 0.05acd *p* > 0.05b
g__Escherichia-Shigella	1.778	2.082	9.630	10.842	*p* > 0.05abcd
g__Bifidobacterium	1.176	2.241	7.376	0.415	*p* > 0.05abc *p* < 0.05d
g__*Lactobacillus*	0.057	0.262	6.419	7.607	*p* < 0.05abc *p* > 0.05d
g__Erysipelatoclostridium	0.125	0.184	6.207	0.817	*p* > 0.05abcd

### Family Level (L5)

At the family level, the gut microbiota of liver transplantation patients contained high levels of the following: *Bacteroidaceae*, Ruminococcaceae, *Lachnospiraceae*, *Prevotellaceae*, and Enterobacteriaceae (BLT group); *Bacteroidaceae*, *Enterococcaceae*, Enterobacteriaceae, *Bifidobacteriaceae* and *Lactobacillaceae* (LT1W group); and Enterobacteriaceae, *Bacteroidaceae*, Ruminococcaceae, *Lactobacillaceae* and *Enterococcaceae* (LT2W group). The CG was characterized by a higher content of *Bacteroidaceae*, Ruminococcaceae, *Lachnospiraceae*, *Prevotellaceae*, and Selenomonadaceae (Figure 4B). *Burkholderiaceae*, Anaerolineaceae, *Aerococcaceae*, P5D1-392, *Mycoplasmataceae*, Steroidobacteraceae, Hydrogenophilaceae, Uncultured, and Bathyarchaeia, appeared alone 1 week after liver transplantation and decreased significantly 2 weeks after surgery. *Deferribacteraceae*, Helicobacteraceae, *Tritrichomonadea*, and Hafniaceae were only present in the CG group, which may be related to liver disease. *Lachnospiraceae*, Monoglobaceae, Oscillospiraceae, and Butyricicoccaceae decreased significantly 1 week after liver transplantation (*p* < 0.05). They began to recover after 2 weeks. *Enterococcaceae* were significantly different between BLT and CG groups, increased sharply 1 week after surgery, and declined 2 weeks later (Table 4).

### Genus Level (L6)

At the genus level, the gut microbiota of the CG and BLT groups contained high levels of *Bacteroides* and *Faecalibacterium*. By contrast, the LT1W and LT2W groups were characterized by a higher content of *Bacteroides*, *Enterococcus*, *Escherichia-Shigella*, *Bifidobacterium*, *Lactobacillus*, and *Erysipelatoclostridium* (Figure 4C). Among the four groups, the bacteria with the greatest differences were *Roseburia*, Erysipelotrichaceae_UCG.003, *Blautia*, *Lachnospira*, *Monoglobus*, *Enterococcus*, *Dorea*, [Eubacterium]_hallii_group, *Intestinibacter*, *Romboutsia*, and [Eubacterium]_eligens_group. Compared with the CG group, the richness of these microorganisms decreased in the BLT group, seemingly due to liver diseases. Following surgery, these microorganisms decreased significantly 1 week later and began to recover after 2 weeks. *Enterococcus* and *Lautropia* appeared immediately after surgery (Table 4).

### Species Diversity Analysis

**FIGURE 5:**
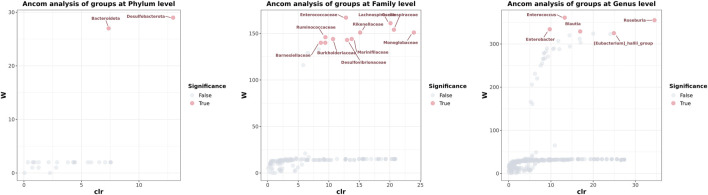
The analysis of composition of microbiomes determined which microorganisms in the samples were significant. Each dot represents a species taxonomic composition. The gray (False) dots indicate that the diversity between groups was not significant, and the red (True) dots indicated that the diversity between groups was significant. The *x*-axis clr represents the relative abundance between groups. The higher the *y*-axis W value, the higher the significance of the species among the groups.

Numerous bacteria, including the phyla Nitrospirota, Chloroflexi, and Crenarchaeota, the families Anaerolineaceae, Aerococcaceae, P5D1-392, *Mycoplasmataceae*, Steroidobacteraceae, Hydrogenophilaceae, and Bathyarchaeia, and the genera *Olsenella*, *Serratia*, and *Enterobacter* were present in the group of patients after liver transplantation only; the phyla Deferribacterota, and Parabasalia, the families *Deferribacteraceae*, Helicobacteraceae, *Tritrichomonadea*, and Hafniaceae, and the genera *Dubosiella*, *Coprobacter*, *Psychrobacter*, and *Mucispirillum* were absent in the diseased individuals and was observed only in healthy individuals (Table 5). Comparison of data of analysis of the taxonomic composition of gut microbiota from patients and CG showed statistically significant differences in the content of some microbial phylum, family, and genus in these groups. These findings suggest that gut microbiota post-transplant are characterized by a decrease in taxonomic diversity and significant differences in the representation of two phyla (Bacteroidetes and Desulfobacterota), 11 families (Barnesiellaceae, Rmuinococcaceae, Burkholderiacrar, *Enterococcaceae*, Desulfovibrionaceae, Marinifilaceae, Rikenellaceae, *Lachnospiraceae*, Oscillosopiraceae, and Monoglobaceae) and five genera (*Enterobacter*, *Enterococcus*, *Blautia*, [Eubacterium]_hallii_group, and *Roseburia*) (Figure 5).

**TABLE 5:** The gut microbiota that occur only before or after liver transplantation.

	CG (%)	BLT (%)	LT1W (%)	LT2W (%)	*p*-value
**Phylum (L2)**					
p__Nitrospirota	0.000	0.000	0.028	0.000	0.345907
p__Chloroflexi	0.000	0.000	0.091	0.000	0.225497
p__Crenarchaeota	0.000	0.000	0.016	0.000	0.345907
p__Deferribacterota	0.152	0.000	0.000	0.000	0.200251
p__Parabasalia	0.015	0.000	0.000	0.000	0.515263
**Family (L5)**					
f__Anaerolineaceae	0.000	0.000	0.073	0.000	0.225497
f__Aerococcaceae	0.000	0.000	0.057	0.000	0.003122
f__P5D1-392	0.000	0.000	0.038	0.000	0.345907
f__*Mycoplasmataceae*	0.000	0.000	0.031	0.000	0.345907
f__Steroidobacteraceae	0.000	0.000	0.027	0.000	0.345907
f__Hydrogenophilaceae	0.000	0.000	0.026	0.000	0.345907
f__Bathyarchaeia	0.000	0.000	0.016	0.000	0.345907
f__*Deferribacteraceae*	0.152	0.000	0.000	0.000	0.200251
f__uncultured	0.046	0.000	0.000	0.000	0.345907
f__Helicobacteraceae	0.042	0.000	0.000	0.000	0.515263
f__*Tritrichomonadea*	0.015	0.000	0.000	0.000	0.515263
f__Hafniaceae	0.012	0.000	0.000	0.000	0.515263
**Genus (L6)**					
g__Dubosiella	0.616	0.000	0.000	0.000	0.515263
g__Coprobacter	0.226	0.000	0.000	0.000	0.002209
g__Psychrobacter	0.221	0.000	0.000	0.000	0.515263
g__[Eubacterium]_ventriosum_group	0.168	0.187	0.000	0.000	2.22E-07
g__Mucispirillum	0.152	0.000	0.000	0.000	0.200251
g__Olsenella	0.000	0.000	0.387	0.000	0.345907
g__Rothia	0.000	0.013	0.336	0.056	0.027494
g__Chloroplast	0.000	0.147	0.303	0.000	0.003862
g__*Serratia*	0.000	0.000	0.000	3.989	0.345907
g__*Enterobacter*	0.000	0.000	0.014	1.270	0.081291

### Differences in Metabolic Pathways Between Groups

**FIGURE 6:**
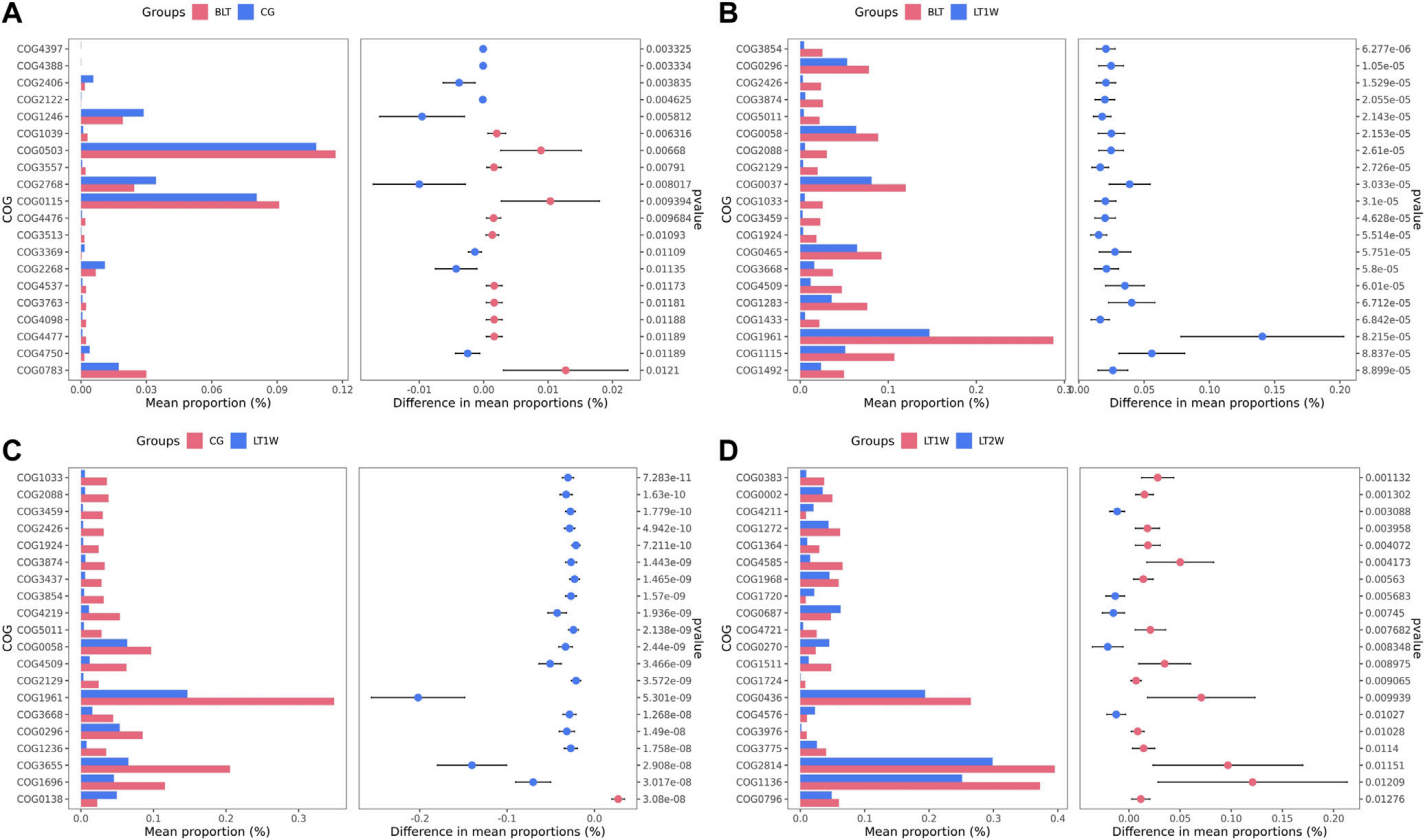
The COG profiles and the components of functional genomics in COG pathways. **(A)**: BLT vs. CG; **(B)**: BLT vs. LT1W; **(C)**: CG vs. LT1W; **(D)**: LT1W vs. LT2W.

We transformed the composition of the OTUs sequences into COG to analyze the differences in the metabolic pathways represented in the gut microbiota among BLT, LT1W, and LT2W group samples. We generated COG profiles and then compared the components of functional genomics in COG pathways. In general, 11 COG functional modules were significantly enriched in the BLT group, nine COG functional modules were significantly less. 19 COG functional modules significantly less, and only one COG functional module was enriched in the LT1W group. On the other hand, compared with the BLT group, 20 functional modules were less represented in the LT1W group. Five COG functional modules were enriched, and 15 COG modules were less represented in the LT2W group than the LT1W group (Figure 6 and Tables 6–9).

**TABLE 6:** The COG of BLT vs. CG.

ID	p-value	Functional description
COG4397	0.003324577	Mu-like prophage major head subunit gpT
COG4388	0.003333848	Mu-like prophage I protein
COG2406	0.003835026	Protein distantly related to bacterial ferritins
COG2122	0.00462527	Uncharacterized conserved protein
COG1246	0.005812124	N-acetylglutamate synthase and related acetyltransferases
COG1039	0.006316055	Ribonuclease HIII
COG0503	0.006680033	Adenine/guanine phosphoribosyltransferases and related PRPP-binding proteins
COG3557	0.007910423	Uncharacterized domain/protein associated with RNAses G and E
COG2768	0.00801657	Uncharacterized Fe-S center protein
COG0115	0.009394288	Branched-chain amino acid aminotransferase/4-amino-4-deoxychorismate lyase
COG4476	0.009683741	Uncharacterized protein conserved in bacteria
COG3513	0.010933294	Uncharacterized protein conserved in bacteria
COG3369	0.011092671	Uncharacterized conserved protein
COG2268	0.0113543	Uncharacterized protein conserved in bacteria
COG4537	0.011729973	Competence protein ComGC
COG3763	0.011809479	Uncharacterized protein conserved in bacteria
COG4098	0.011884764	Superfamily II DNA/RNA helicase required for DNA uptake (late competence protein)
COG4477	0.011889677	Negative regulator of septation ring formation
COG4750	0.011893088	CTP:phosphocholine cytidylyltransferase involved in choline phosphorylation for cell surface LPS epitopes
COG0783	0.01209744	DNA-binding ferritin-like protein (oxidative damage protectant)

**TABLE 7:** The COG of BLT vs. LT1W.

ID	*p*-value	Functional description
COG3854	6.27678E-06	Stage III sporulation protein SpoIIIAA
COG0296	1.04985E-05	1,4-alpha-glucan branching enzyme
COG2426	1.52853E-05	Predicted membrane protein
COG3874	2.0547E-05	Uncharacterized conserved protein
COG5011	2.14315E-05	Uncharacterized protein conserved in bacteria
COG0058	2.15279E-05	Glucan phosphorylase
COG2088	2.61E-05	Uncharacterized protein, involved in the regulation of septum location
COG2129	2.72612E-05	Predicted phosphoesterases, related to the Icc protein
COG0037	3.03301E-05	Predicted ATPase of the PP-loop superfamily implicated in cell cycle control
COG1033	3.09983E-05	Predicted exporters of the RND superfamily
COG3459	4.62774E-05	Cellobiose phosphorylase
COG1924	5.51436E-05	Activator of 2-hydroxyglutaryl-CoA dehydratase (HSP70-class ATPase domain)
COG0465	5.75095E-05	ATP-dependent Zn proteases
COG3668	5.80032E-05	Plasmid stabilization system protein
COG4509	6.00954E-05	Uncharacterized protein conserved in bacteria
COG1283	6.71153E-05	Na+/phosphate symporter
COG1433	6.84207E-05	Uncharacterized conserved protein
COG1961	8.21533E-05	Site-specific recombinases, DNA invertase Pin homologs
COG1115	8.83694E-05	Na+/alanine symporter
COG1492	8.89871E-05	Cobyric acid synthase

**TABLE 8:** The COG of LT1W vs. CG.

ID	*p*-value	Functional description
COG1033	7.28286E-11	Predicted exporters of the RND superfamily
COG2088	1.62982E-10	Uncharacterized protein, involved in the regulation of septum location
COG3459	1.77916E-10	Cellobiose phosphorylase
COG2426	4.94246E-10	Predicted membrane protein
COG1924	7.21105E-10	Activator of 2-hydroxyglutaryl-CoA dehydratase (HSP70-class ATPase domain)
COG3874	1.44265E-09	Uncharacterized conserved protein
COG3437	1.46492E-09	Response regulator containing a CheY-like receiver domain and an HD-GYP domain
COG3854	1.56993E-09	Stage III sporulation protein SpoIIIAA
COG4219	1.93588E-09	Antirepressor regulating drug resistance, predicted signal transduction N-terminal membrane component
COG5011	2.13803E-09	Uncharacterized protein conserved in bacteria
COG0058	2.43995E-09	Glucan phosphorylase
COG4509	3.4655E-09	Uncharacterized protein conserved in bacteria
COG2129	3.57172E-09	Predicted phosphoesterases, related to the Icc protein
COG1961	5.30064E-09	Site-specific recombinases, DNA invertase Pin homologs
COG3668	1.26795E-08	Plasmid stabilization system protein
COG0296	1.49041E-08	1,4-alpha-glucan branching enzyme
COG1236	1.75778E-08	Predicted exonuclease of the beta-lactamase fold involved in RNA processing
COG3655	2.90768E-08	Predicted transcriptional regulator
COG1696	3.01671E-08	Predicted membrane protein involved in D-alanine export
COG0138	3.08025E-08	AICAR transformylase/IMP cyclohydrolase PurH (only IMP cyclohydrolase domain in Aful)

**TABLE 9:** The COG of LT1W vs. LT2W.

ID	*p*-value	Functional description
COG0383	0.001132206	Alpha-mannosidase
COG0002	0.001302019	Acetylglutamate semialdehyde dehydrogenase
COG4211	0.003087535	ABC-type glucose/galactose transport system, permease component
COG1272	0.003958178	Predicted membrane protein, hemolysin III homolog
COG1364	0.004072359	N-acetylglutamate synthase (N-acetylornithine aminotransferase)
COG4585	0.004172932	Signal transduction histidine kinase
COG1968	0.005630082	Uncharacterized bacitracin resistance protein
COG1720	0.005683167	Uncharacterized conserved protein
COG0687	0.007449622	Spermidine/putrescine-binding periplasmic protein
COG4721	0.007681902	Predicted membrane protein
COG0270	0.008347795	Site-specific DNA methylase
COG1511	0.008975009	Predicted membrane protein
COG1724	0.009064968	Predicted periplasmic or secreted lipoprotein
COG0436	0.009938515	Aspartate/tyrosine/aromatic aminotransferase
COG4576	0.010274524	Carbon dioxide concentrating mechanism/carboxysome shell protein
COG3976	0.010283793	Uncharacterized protein conserved in bacteria
COG3775	0.011395885	Phosphotransferase system, galactitol-specific IIC component
COG2814	0.011505916	Arabinose efflux permease
COG1136	0.012085984	ABC-type antimicrobial peptide transport system, ATPase component
COG0796	0.01276487	Glutamate racemase

## Discussion

Overall, the healthy gut microbiota is dominated by the phyla Firmicutes and Bacteroidetes, followed by Proteobacteria and Actinobacteria. Core microbial diversity and the ratio of Firmicutes and Bacteroidetes are general health indicators. Traditionally, the Firmicutes to Bacteroidetes (F/B) ratio is implicated in predisposition to disease states (Ley et al., 2006). The F/B ratio in the LT1W and LT2W groups were higher than the BLT group. The F/B of the LT1W group was the largest of the four groups, suggesting patients were most susceptible to infection during the first week after liver transplantation. At this point, the structure of intestinal bacteria of the patients had undergone significant changes.

Obligate anaerobic bacteria (such as the phyla Firmicutes and Bacteroidetes) encode various enzymes for hydrolyzing complex carbohydrates not digestible by the host, such as resistant starch and fiber. Genera such as *Lactobacillus* and *Bifidobacterium* specialize in oligosaccharide fermentation, utilizing galactooligosaccharides, fructooligosaccharides, and the polysaccharide inulin (Sims et al., 2014). Carbohydrate fermentation by anaerobes provides the host with essential short-chain fatty acids (SCFAs) such as acetate, propionate, and butyrate (Andoh, 2016). It is known that antibiotics deplete microbes that ferment essential SCFAs such as butyrate, which are typically responsible for maintaining microbial homeostasis. The lack of butyrate silences metabolic signaling in the gut. Mitochondrial Beta-oxidation in colonocytes becomes disabled, resulting in oxygen transfer, which freely diffuses across cell membranes from the blood to the gut lumen. Oxygen in the colon then allows for pathogenic facultative anaerobes such as *E. coli* to outcompete the benign obligate anaerobes that characterize a healthy gut (Winter et al., 2013; Byndloss et al., 2017; Wassenaar, 2016).

Facultative anaerobes, including Proteobacteria, further affect nutrition by catabolizing SCFAs present in the lumen (Litvak et al., 2018). Microbial homeostasis is typically maintained by peroxisome proliferator-activated receptor gamma (PPAR-γ). PPAR-γ is a nuclear receptor activated by butyrate and other ligands, is found in adipocytes and colonocytes, and is responsible for activating genes involved in glucose and lipid metabolism (Winter et al., 2014). Dysbiosis of the gut microbiota occurred in the patients after surgery. Blooms of facultative anaerobes, particularly Enterobacteriaceae, are associated with inflammatory conditions in the gut. However, the healthy colon is almost entirely anaerobic, and there, obligate anaerobes rely on the fermentation of carbohydrates and amino acids to generate energy. By-products of this process include the SCFAs, which are thought to play essential roles in maintaining epithelial integrity and supporting an anti-inflammatory state (Goverse et al., 2017; Desai et al., 2016; Wu et al., 2017). Enterobacteriaceae multiply in large numbers in patients, suggesting that the intestinal permeability had been broken. There is evidence that inflammation is associated with increased Enterobacteriaceae abundance (Lopez et al., 2012). Some of these significant changes were observed in bacterial species belonging to *Enterococcus*, *Klebsiella*, and *Enterobacter*, seen in patients’ intestines (Figure 5; Supplementary Figure S2). These are members of the ESKAPE group (*Enterococcus faecium*, *Staphylococcus aureus*, *Klebisiella pneumoniae*, *Acinetobacter baumannii*, *Pseudomonas aeruginosa* and *Enterobacter* spp. are collectively knowen as ESKAPE group pathogen), described as the leading cause of resistant nosocomial infections (Davin-Regli et al., 2019).

Indeed, loss of balance in microbial population and function, or dysbiosis, provokes the disruption of the intestinal barrier tight junctions. This morphological alteration leads to increased intestinal permeability (also known as leaky gut) and an increment in the portal influx of bacteria or their products to the liver (Albillos et al., 2020). An increment in permeability in the gut and the translocation of bacteria facilitate microbial metabolites entering the liver, leading to impairment of bile acid metabolism and promoting systemic inflammation and gut dysmotility. Bile acids in the gut can maintain the balance of intestinal microbiota by controlling the pH of the intestinal environment and inhibiting the growth of pathogens.

Our findings suggest that the operation of liver transplantation and the use of antibiotics substantially alter the balance of microecology in patients’ intestines. The number and proportion of probiotics decreased significantly, and the number and proportion of pathogenic bacteria showed a considerable upward trend. The ratio of Bifidobacterium to Enterobacteriaceae (B/E rate) includes the primary obligate anaerobic beneficial bacteria and the facultative anaerobic conditional pathogens, which constitute the colonization resistance of the gut. The B/E rate can be used to represent intestinal microbiota imbalance. B/E <1 indicates dysbiosis of the gut microbiota, and the lower B/E, the greater the degree of dysbiosis of the gut microbiota. Compared with healthy people (CG, B/E = 0.991), due to the dominant bacteria in patients with chronic liver disease (BLT, B/E = 0.921) lose their advantages in the gut, the antagonistic effect on the inferior bacteria is reduced, and the inferior bacteria produce a large amount of endotoxin during the process (Islam et al., 2011). The decline of liver function affects the production of bile acids. The B/E of LT1W and LT2W groups were 0.921 and 0.481, respectively. By contrast, gastrointestinal dysfunction and intestinal peristalsis often occur after liver transplantation, creating an intestinal environment conducive to the reproduction of conditionally pathogenic bacteria.

It is noteworthy that, at the genus level, *Blautia* almost disappeared after liver transplantation (Figure 5). As a genus of the *Lachnospiraceae* family, *Blautia* has been of particular interest because of its contribution to alleviating inflammatory diseases and metabolic diseases and its antibacterial activity against specific microorganisms (Kalyana et al., 2018). *Blautia* is a dominant genus in the intestinal microbiota, and there are significant correlations with host physiological dysfunctions such as obesity, diabetes, cancer, and various inflammatory diseases. Liu et al. speculated that the ability to produce bacteriocins provides *Blautia* with the potential to inhibit the colonization of pathogenic bacteria in the gut, and it can also affect the composition of intestinal microbiota. *Blautia* inhibits the proliferation of *C.perfringens* and vancomycin-resistant *Enterococci*, which makes it possible to become potential probiotics and exert probiotic functions (Liu et al., 2021).

The abundance of the phylum Proteobacteria was markedly high, and its absence and low abundance of signature genera such as *Bacteroides*, *Prevotella*, and *Ruminococcus* suggest an unhealthy gut microbiota in patients in the first week after liver transplantation (Hollister et al., 2014). Even if an organ preservation solution can effectively ensure the survival of the liver, it is still difficult to avoid hypoxia and mechanical damage to the liver. The degree of transaminase and disturbance of bile acid secretion after surgery also aggravates the reproduction of pathogenic bacteria in the gut.

We generated COG profiles and compared the representations of COG functional pathways. Compared with healthy samples, the functional modules related to protein distantly related to bacterial ferritins, N-acetylglutamate synthase and related acetyltransferases, protein synthesis, and phosphocholine cytidylyltransferase involved in choline phosphorylation for cell surface lipopolysaccharide epitopes in patients before surgery were significantly reduced. Notably, 19 of the top 20 functional modules decreased significantly in patients 1 week after liver transplantation, including cellulose hydrolysis, glucose metabolism, DNA synthesis, and RNA transcription. In the LT1W group, the top 20 functional modules were less represented than the BLT group: COG1492 is a member of a metabolic pathway for the synthesis of vitamin 12; COG1961 is related to DNA synthesis; COG1115 is related to alanine; and COG0296/COG0058/COG2129 is related to glucose metabolism. Five COG functional modules were enriched, 15 COG modules were less represented in the LT2W group than the LT1W group: COG2814/COG1136 is related to biological transport processes; COG0436 is related to amino acid metabolism; COG0270 is related to cell proliferation; and COG0383 plays a decisive role in the synthesis of protein and folding in correct conformations (Figure 6; Tables 6–9).

Live *Bifidobacterium* capsules are commercially available; these probiotics colonize the gut, antagonize the growth of pathogenic bacteria in the gut, form a biofilm barrier, inhibit the growth of pathogenic bacteria, reduce intestinal endotoxin and bacterial translocation, reduce the damage of intestinal mucosal epithelium, reduce its permeability, and reduce or delay its atrophy (Holma et al., 2014; Baek et al., 2014; Gianotti et al., 2010; Horvat et al., 2010; Rafter et al., 2007; Zhu et al., 2012). *Bifidobacterium* also accelerates the decomposition and absorption of nutrients in the gastrointestinal tract, producing many acidic substances, acidifying the intestinal cavity, accelerating the excretion of endotoxin, and reducing the damage of intestinal mucosa (Li et al., 2010; Gorska et al., 2009; Ohland et al., 2010).

The active use of liver bacteria preparations or fecal bacteria transplantation in the perioperative period of liver transplantation patients is expected to improve patients’ intestinal microecology, reduce postoperative complications, and accelerate postoperative recovery.

## Conclusion

We characterized the gut microbiome in patients during the perioperative liver transplantation period and compared the diversity, richness, and compositional variation of the gut microbiome between the healthy control and patient groups. We observed an altered microbial composition post-liver transplantation, suggesting a distinct signature of microbiota associated with the procedure in liver transplant patients. Although numerous bacterial species were particularly present or absent in patient samples, we could not effectively compare the species-level composition due to low sequencing depth and small numbers of samples. Although this is a preliminary study to explore the liver transplantation gut microbiome compared to the healthy gut microbiome of the population, it is subject to limitations resulting from low sample numbers and sequencing depth. Most participants were sampled only once; a time-series monitoring of multiple samples at various time points and a more significant number of participants would provide more insights that might have been missed due to low sequencing depth. Nevertheless, the present study is significant because it provides the scientific community with a glimpse of the signature microbiota associated with liver transplantation individuals in Shanxi province. Furthermore, it provides a road map for future studies. We believe that this signature of liver transplantation individuals in the Chinese population will aid future studies.

## Data Availability

The datasets presented in this study can be found in online repositories. The names of the repository/repositories and accession number(s) can be found below: NCBI with BioProject ID PRJNA811387.
