# Supplementary figures and images for: The Gut Microbiota in Liver Transplantation Recipients During the Perioperative Period
Source: Front Physiol. 2022 Apr 1;13:854017. doi: 10.3389/fphys.2022.854017 (PMC9075733; doi:10.3389/fphys.2022.854017)

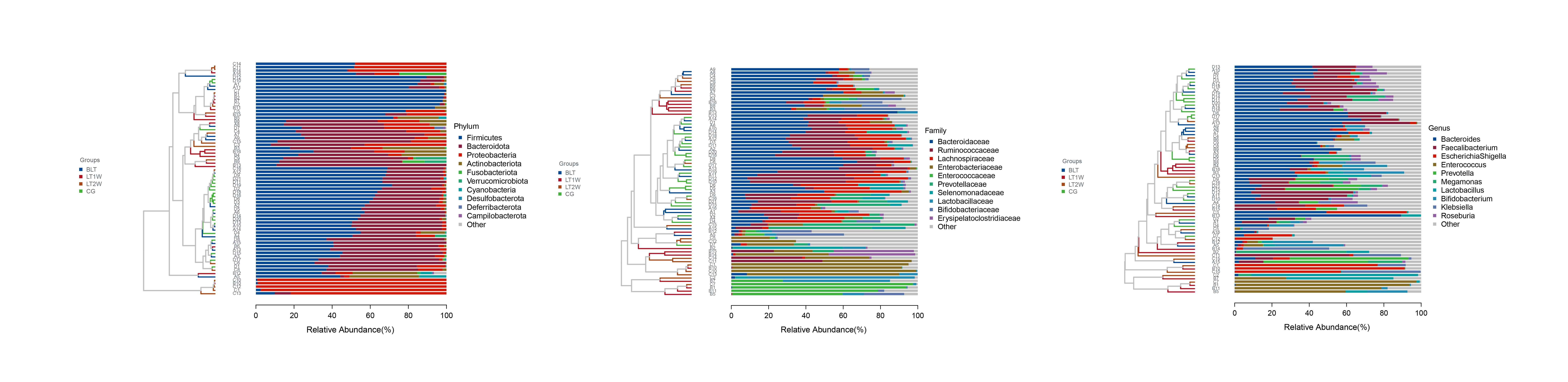

Supplement: Supplementary file 1 [file Image1.JPEG]

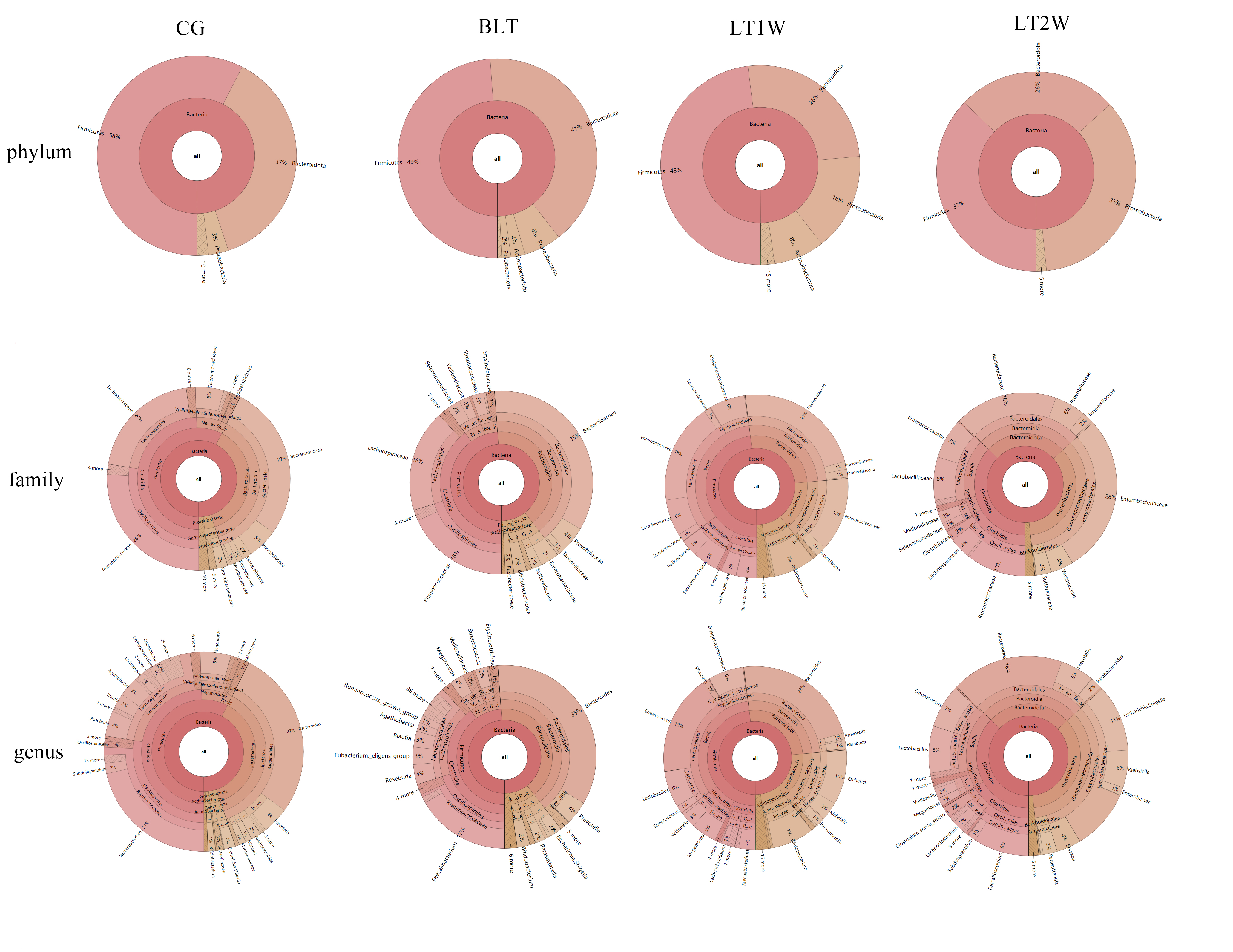

Supplement: Supplementary file 2 [file Image2.JPEG]
